# MicroRNAs derived from the insect virus *Hz*NV-1 promote lytic infection by suppressing histone methylation

**DOI:** 10.1038/s41598-018-35782-w

**Published:** 2018-12-13

**Authors:** Pei-Chi Wu, Yu-Hsien Lin, Tsai-Chin Wu, Song-Tay Lee, Carol-P. Wu, Yuan Chang, Yueh-Lung Wu

**Affiliations:** 1 Department of Entomology, National Taiwan University, Taipei, 106 Taiwan; 2 Institute of Entomology, Biology Centre CAS, Ceske Budejovice, Czech Republic; 3 Faculty of Science, University of South Bohemia, Ceske Budejovice, Czech Republic; 4 Department of Biotechnology, Southern Taiwan University of Technology, Tainan, 710 Taiwan

## Abstract

*Heliothis zea* nudivirus-1 (*Hz*NV-1) is an insect virus that can induce both lytic and latent infections in various insect cell lines. During latent infection, several microRNAs (miRNAs) are produced from persistency-associated gene 1 (*pag1*) as the only detectable *Hz*NV-1 transcript. Previous studies have shown that the *pag1* gene suppresses the immediate-early gene *hhi1* and promotes host switching into a latent infection via miRNAs derived from *pag1*. Although other functions of the miRNAs derived from *pag1* have not yet been elucidated, several studies have suggested that miRNAs encoded from latency-associated genes can regulate histone-associated enzymes. Because *pag1* is a noncoding transcript, it potentially regulates host chromatin structure through miRNAs upon infection. Nevertheless, the exact mechanism by which *pag1* alters viral infections remains unknown. In this study, we found that the *pag1*-encoded miRNA miR-420 suppresses expression of the histone modification-associated enzyme *su(var)3–9*. Therefore, this miRNA causes histone modification to promote *Hz*NV-1 infection. These results suggest that *Hz*NV-1 may directly influence epigenetic regulation in host cells through interactions with *pag1* miRNAs to promote lytic infection. This study provides us with a better understanding of both the *Hz*NV-1 infection pathway and the relationship between viral miRNAs and epigenetic regulation.

## Introduction

*Heliothis zea* nudivirus-1 (*Hz*NV-1), an insect virus with a broad host range^1^, has a circular double-stranded DNA genome that encodes approximately 154 open reading frames^2^. This virus was previously classified in the *Baculoviridae* family based on similarity with baculovirus in regard to structure and replication mode. However, *Hz*NV-1 was later placed in the family *Nudiviridae* due to its lack of occlusion bodies and higher genomic homology with nudiviruses than baculovirus^3^. We previously reported that *Hz*NV-1 produces more than 100 transcripts in productive infection, with the immediate-early gene *hhi1* generating high levels of a 6.2-kb transcript^4^. It is known that *hhi1* can serve as a transcriptional activator and that it is involved in viral reactivation from latently infected cells^5^. In contrast to the expression pattern of most immediate-early (IE) genes of baculovirus, expression of *hhi1* was recently shown to require the assistance of viral factors^5^. Only one gene transcript, named persistency-associated transcript (PAT1), is detectable from persistency-associated gene 1 (*pag1*) during latent viral infection, and this unique noncoding RNA was found to be responsible for, or at least involved in, the establishment of latent viral infection^4,6^.

The *pag1* transcript comprises a series of microRNAs (miRNAs), with no sequences translated into protein. Wu *et al*. observed that several miRNAs encoded by *pag1* inhibit *hhi1* expression, resulting in a higher proportion of latent than lytic infection^7^. However, the functions of other miRNAs have not been elucidated to date. Small RNA molecules (approximately 22 nucleotides) that form stem-loop structures and function as silencers, miRNAs downregulate gene expression, in some cases by targeting the promoter of messenger RNAs (mRNAs)^8,9^. Many miRNA functions have been identified in insects^10^. For instance, miRNAs have been discovered to be involved in metabolism^11,12^, social behavior^13^ and interactions between viruses and hosts^14,15^. Viruses, including *Heliothis virescens* ascovirus (*Hv*AV)^16^ and West Nile virus (WNV), have also been observed to employ their own miRNAs to regulate viral replication^17^.

Epigenetic regulation involves histone modification^18^, DNA methylation/acetylation^19^, and miRNA expression^20^. Recently, Kaposi’s sarcoma-associated herpesvirus (KSHV) has been shown to regulate its lytic/latent cycle through histone modification^21^, and active or repressive histone markers are reportedly distributed across the entire latent KSHV genome, suggesting that this phenomenon is dynamic^22,23^. Histone H3-lysine^24^ trimethylation (H3K27me3), the dominant repressive marker, covers the majority of the KSHV genome and suppresses lytic gene expression. In addition to KSHV, almost all human herpesviruses (HSV, KHSV and HCMV) encode viral miRNAs that inhibit IE gene expression to regulate lytic and latent infection^25,26^. Methylation and acetylation of histone tails are carried out by DNA methyltransferases (DNMTs) and histone acetyltransferases (HATs), respectively^24,27^. There are two forms of chromatin: heterochromatin and euchromatin. The former is defined as condensed chromatin in which transcription is blocked, whereas euchromatin is defined as lightly packed, unstainable chromatin that is often present during active transcription^18,28^. Histone-lysine N-methyltransferase *Su(var)* function is related to heterochromatin with position-effect variegation (PEV)^29,30^, and one member of the su(var) group, *su(var)3–9*, that causes histone H3-lysine^9^ (H3K9) methylation has been extensively studied in *Drosophila* and mammals^31,32^. Furthermore, *su(var)* is present in *Spodoptera frugiperda* and two additional *Spodoptera* species and is associated with the silencing of viral gene expression through interactions with epigenetic factors^33^. Another gene, *tip60*, encoding a HAT is also associated with pathogenic infection in Lepidoptera insects^34^.

HSVs have been found to modify host histone tails during virus infection. As these reports primarily focused on the promoters of lytic and latent genes and assessed histone methylation associated with gene suppression^8^, sites with higher levels of methylation during the latent stage have been well studied^8,35^. In contrast, studies of epigenetic regulation in viruses are still limited. Therefore, we used the *pag1* gene, which produces the only detectable gene transcript during latent *Hz*NV-1 infection, as a model system to study whether *pag1*-encoded miRNAs directly affect host histone modification. The results of this study suggest that this is not the case. We chose *Hz*NV-1, which has features similar to those of HSVs, as our model system. We first observed a decline in H3K9me3 levels, in contrast to other reports, whereas acetylation levels were significantly enhanced by the presence of the *pag1* gene. We next identified two histone transferases that might be responsible for these phenomena, *su(var)3–9* and *tip60*. We demonstrated that *su(var)3–9* was downregulated by a miRNA encoded from *pag1*: miR-420. We found evidence supporting that *Hz*NV-1 uses its latent infection-associated miRNAs to control host histone modification and promote lytic infection. We propose a model that accounts for the latent infection of insect viruses and can also be applied to mammalian viruses.

## Results

### Histone modification detected at different time points after *pag1* transfection

**Figure 1:**
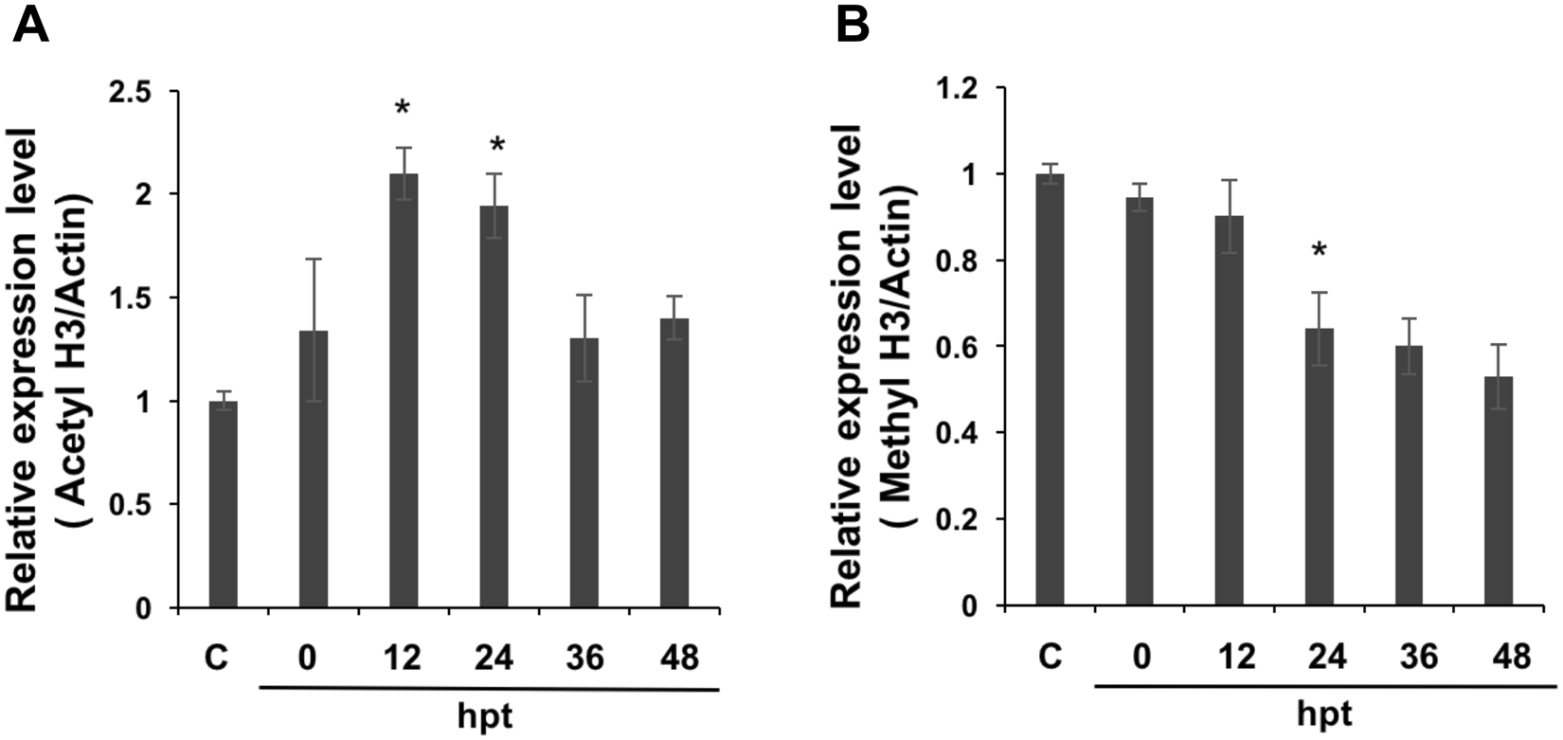
Western blot detection of histone 3 acetylation/methylation levels after *pag1* transfection. (**A**) Acetylation levels of H3 were detected with an anti-acetyl-H3 antibody. (**B**) Methylation levels were detected with an anti-trimethyl-H3K9 antibody. Samples were extracted using RIPA buffer. The band areas were quantified to generate the upper bar charts. The samples were normalized to actin, and the control group was set as 1. Mean and standard deviation (SD) values are shown, and P values were calculated using Student’s t-test (*P < 0.05). All experiments were performed with three replicates.

As previous studies have shown that *pag1* regulated viral gene expression and influenced the type of infection^5^, the association between the *pag1* gene and host histone status was therefore examined. Since the amino-terminal tail of histone 3 has been studied thoroughly in recent years, the histone 3 modification pattern was chosen as a reference for examining changes in histone modifications caused by *pag1* using acetylation H3 and trimethylated H3K9 antibodies (Fig. 1). After *pag1* was transfected into cells, strong acetylation was observed up to 36 hour post-transfection (hpt). The acetylation levels of *pag1* samples were higher than those of the control group throughout the experiment and peaked at 24 hours post-transfection (Fig. 1A); conversely, methylation in host cells decreased after transfection (Fig. 1B). These data suggest that the *pag1* gene may encode factor(s), which would upregulate gene expression. Although previous studies have shown that methylation levels of lytic gene transcripts may increase during latent infection, the distribution of epigenetic markers is suggested to be dynamic across the entire host genome during the latent stage.

**Figure 2:**
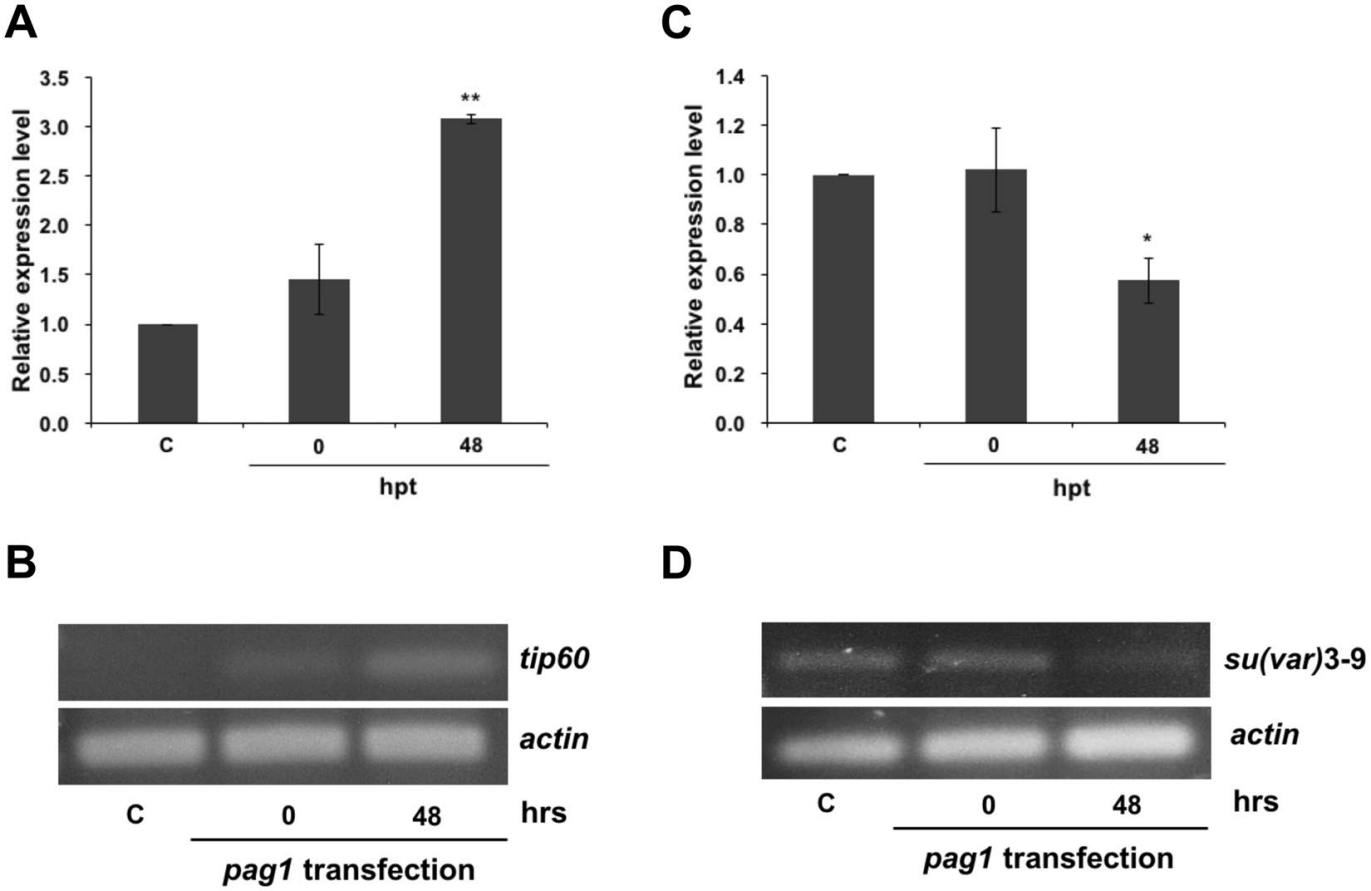
Real-time PCR and RT-PCR detection of *tip60* and *su(var)3–9* expression levels. (**A**) HAT *tip60* gene expression levels were detected at 0 and 48 hours after *pag1* transfection. (**B**) RT-PCR analysis of *tip60* expression levels at 48 hpt. (**C**) *Su(var)3–9* gene expression levels were detected at 0 and 48 hours after *pag1* transfection by RT-qPCR. (**D**) RT-PCR analysis of *su(var)3–9* expression levels at 48 hpt. All results were normalized to those for *actin*. At least three repetitions were conducted for each group. P values were calculated using Student’s t-test (*P < 0.05; **P < 0.005).

To further confirm that *pag1* was able to alter host histone modifications, two histone-associated enzymes, HATs *tip60* and *su(var)3–9*, were chosen to explore the role of the *pag1* gene in the histone modification pathway. The expression levels of HAT *tip60* and *su(var)3–9* at 0 to 48 hours post *pag1* transfection were measured. Previous studies have revealed that HAT *tip60* is associated with H3 acetylation, and the result here showed that *tip60* expression was increased upon *pag1* transfection (Fig. 2B), which was in agreement with the western blot results (Fig. 1B) and previous findings, suggesting that acetylation increased after *pag1* transfection. The acetylation levels after *pag1* transfection were 3-fold higher than those in the control group at 48 hours post-transfection (Fig. 2A,B), and the presence of *pag1* suppressed *su(var)3–9* expression by 50% (Fig. 2C,D). It is worth noting that *pag1* does not encode a protein, but instead miRNAs are produced. Several studies have suggested that the miRNAs produced from latent-associated genes are mostly involved in regulating histone-associated enzymes^36^. *Su(var)3–9* trimethylates H3K9, and it has been identified in different animals^33^, though similar mechanisms in insects have not yet been identified. Therefore, miRNAs may be crucial for the mechanism by which *Hz*NV-1 affects these two histone transferases.

### Predicted and identified miRNAs produced by the *pag1* transcript

**Figure 3:**
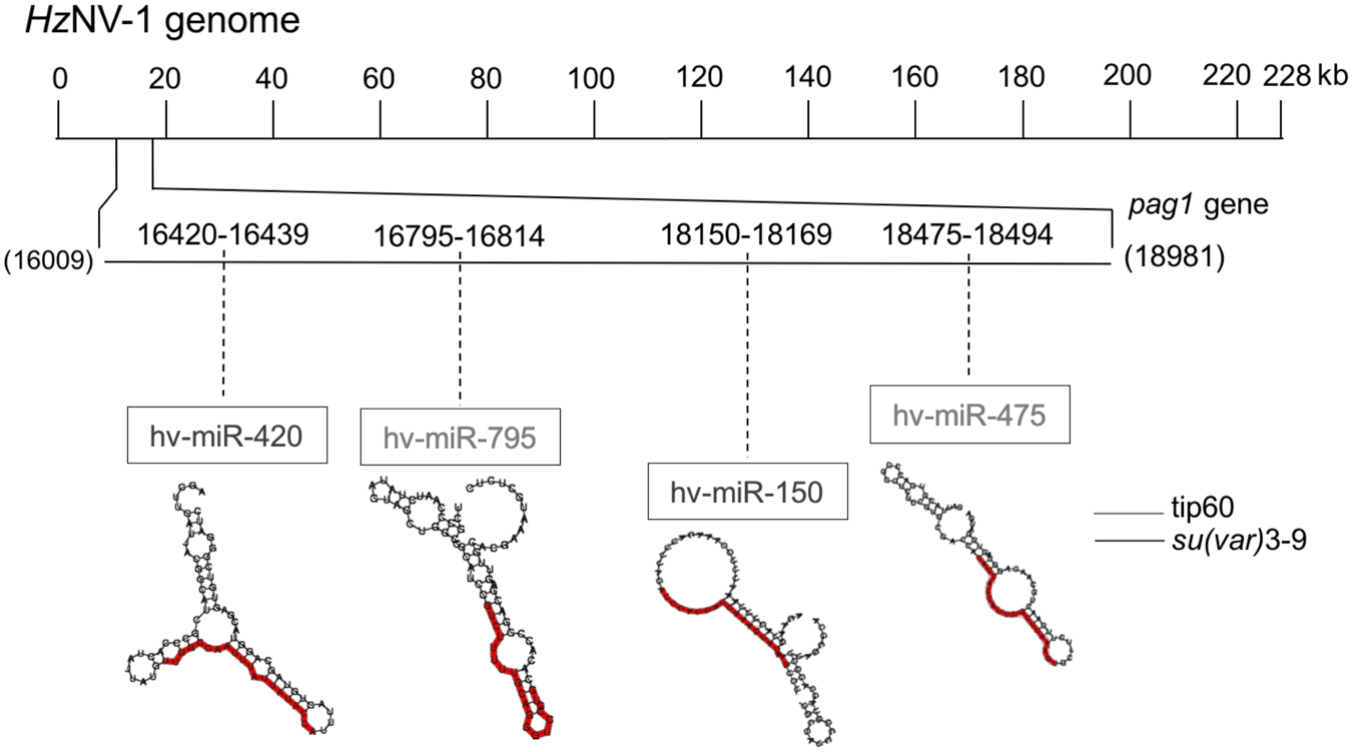
Mapping of *pag1* miRNAs that target *tip60* or *su(var) 3–9* in the *Hz*NV-1 genome. The complete sequence of the *Hz*NV-1 genome is shown in the upper portion. The *pag1* gene is located from nt 16,009 to 18,981. The gray lines indicate miRNAs targeting the *tip60* gene. The black lines represent miRNAs targeting the *su(var)3–9* gene. The predicted structures of the miRNAs are displayed in Table 1.

**Figure 4:**
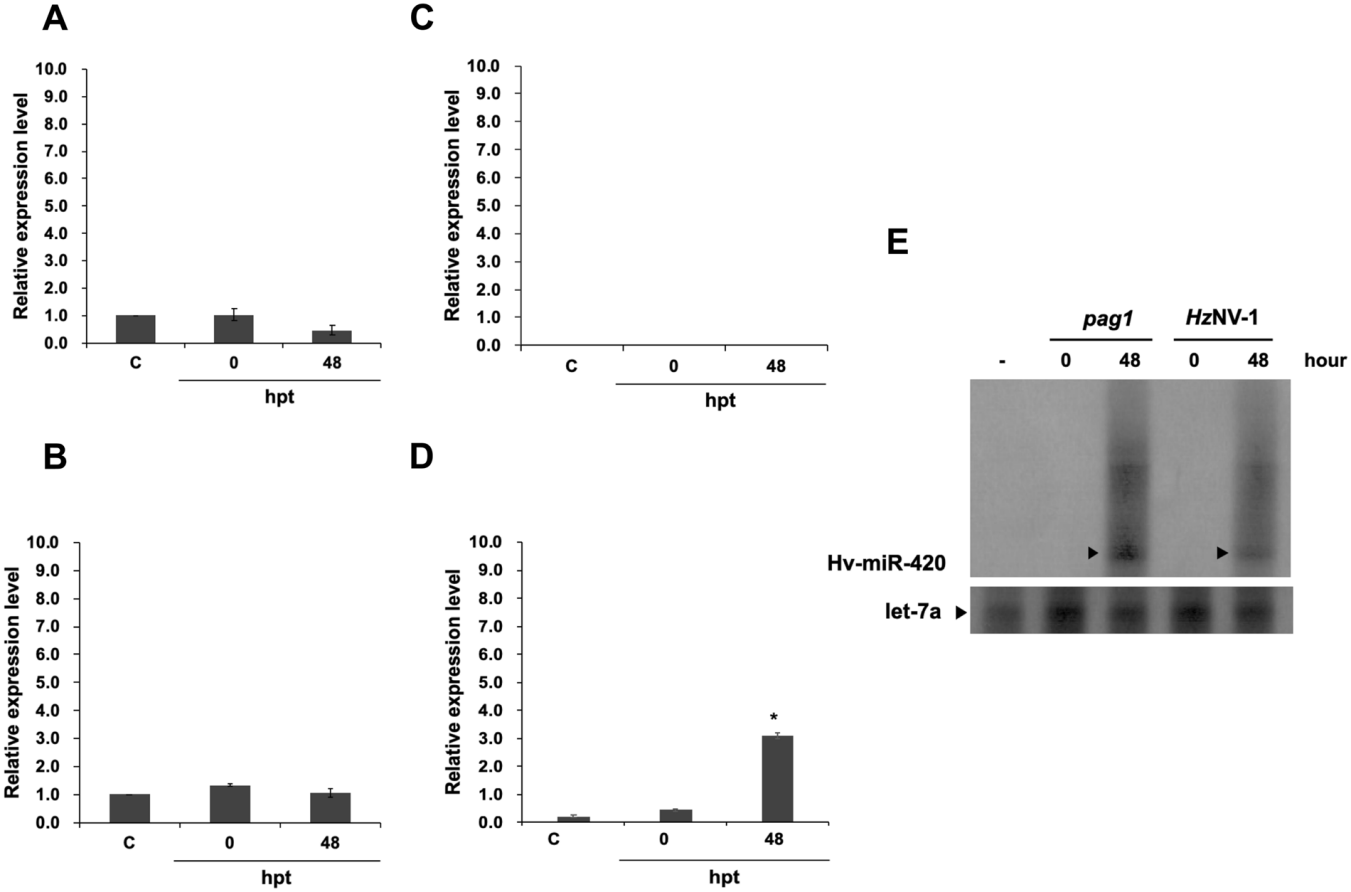
Stem-loop RT-qPCR confirmation of miRNA expression after *pag1* transfection. (**A**) Hv-miR-795 levels decreased after *pag1* transfection. (**B**) Hv-miR-475 was not affected by *pag1* in Sf-21 cells. (**C**) Hv-miR-150 was not detected at any time point. (**D**) Hv-miR-420 was identified after *pag1* transfection. (**E**) Small RNAs harvested from *pag1*-transfected cells and *Hz*NV-1 productively infected cells were analyzed by northern blotting using probes against predicted *Hz*NV-1 miR-420 (top panels) or let-7a miRNA as a positive control (bottom panels). At least three repetitions were conducted for each group. P values were calculated using Student’s t-test (*P < 0.05).

According to the NCBI database, the complete genome of *Hz*NV-1 is approximately 228 kb, and the *pag1* transcript is approximately 4 kb. To identify miRNAs that target the histone transferases encoded by *tip60* and *su(var)3–9*, *pag1*-encoded miRNAs were mapped onto these genes. Prediction was performed by Vir-Mir and based on mapping scores and structures. Four candidate miRNAs (Hv-miR-420, Hv-miR-795, Hv-miR-150, Hv-miR-475) were selected for further evaluation (Fig. 3). The sequences of the miRNAs are listed in Table 1. To verify that the predicted miRNAs are actually encoded and generated from *pag1*, stem-loop RT-qPCR was used to measure these candidate miRNAs at different time points after *pag1* transfection in Sf-21 cells. Among these miRNAs, Hv-miR-795 (Fig. 4A) and Hv-miR-475 (Fig. 4B) were detected in both *pag1-*transfected cells and in untransfected cells, suggesting that host cells also expressed these 2 miRNAs. Hv-miR-150 was undetectable in both groups, indicating that Hv-miR-150 is a mock miRNA (Fig. 4C). Hv-miR-420 was the only candidate miRNA whose expressed was significantly increased after *pag1* transfection (Fig. 4D). To further confirm the results of stem-loop PCR, northern blot analysis was performed, showing detection of Hv-miR-420 in both *pag1*-transfected and *Hz*NV-1-infected cells (Fig. 4E). These results showed that the expression level of Hv-miR-420 was significantly increased in *pag1*-transfected and *Hz*NV-1-infected cells.

**Table 1:** The predicted miRNAs produced by the *pag1* gene.

Target	Name	Location	Sequence
*Su(var)3–9*	Hv-miR-420	16420–16439	5′-UUGGCCAAUUUAUUAUACCA-3′
	Hv-miR-150	18150–18169	5′-AUCCAUGAUUAAAGUACAAG-3′
*tip60*	Hv-miR-795	16795–16814	5′-GUCUUUUUGCAGGGUCCGUG-3′
	Hv-miR-475	18475–18494	5′-CACCACCAUUCGUUUAAAGU-3′

### Hv-miR-420 suppresses *su(var)3–9* expression

**Figure 5:**
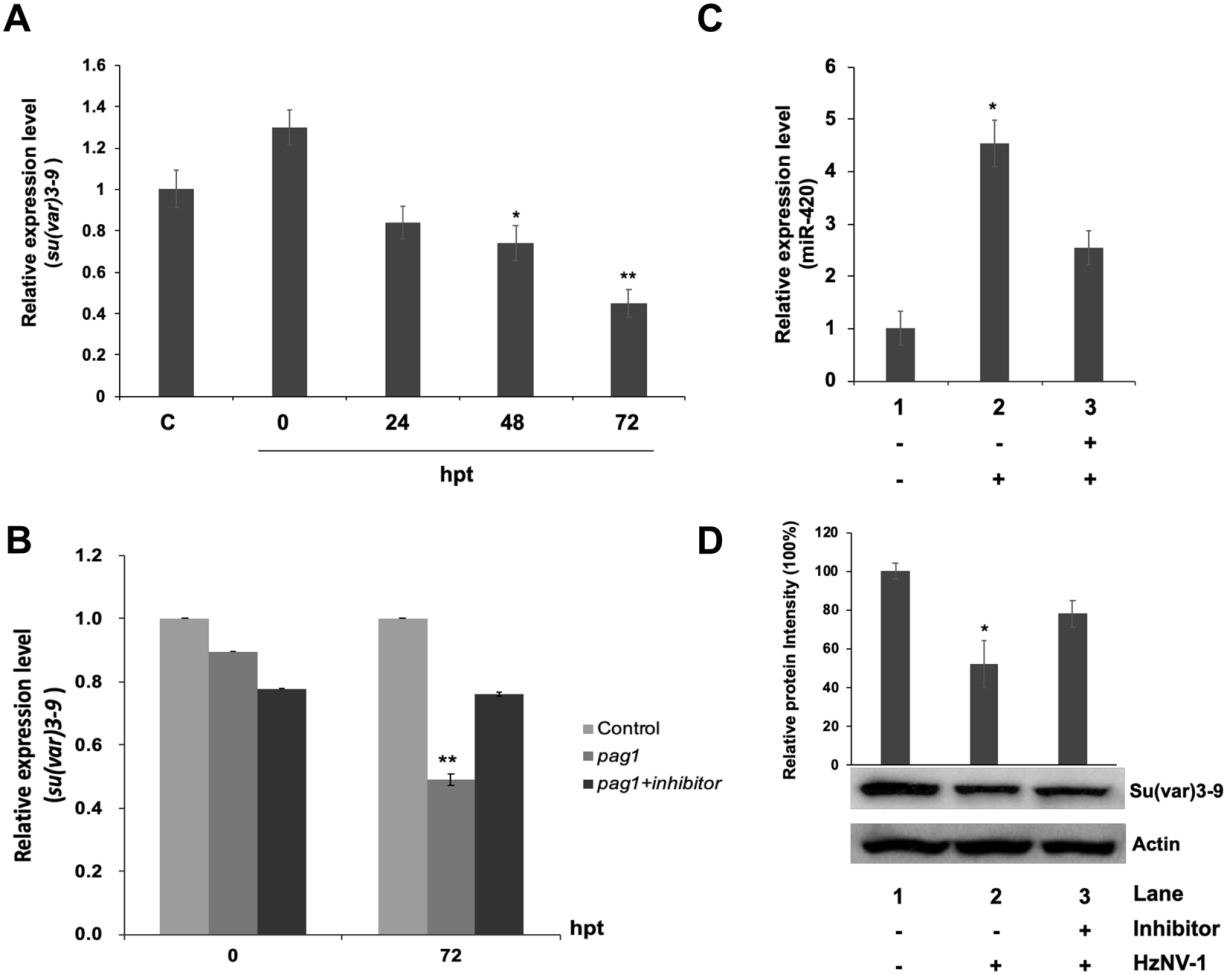
miR-420 regulates *su(var)3–9* expression levels in Sf-21 cells. (**A**) *Su(var)3–9* expression was downregulated by miR-420 and 60% inhibited at 72 hours post-transfection. (**B**) The miR-420 inhibitor rescued *su(var)3–9* expression at 72 hours. (**C**) Stem-loop RT-qPCR confirmed miR-420 expression after *Hz*NV-1 infection with or without the miR-420 inhibitor. (**D**) Su(var)3–9 expression was detected at 48 hours after *Hz*NV-1 infection with or without the miR-420 inhibitor by western blotting. All results were normalized to the CT value of *actin*, and at least three repetitions were performed. The control group expression level was set to 1 or 100%. P values were calculated using Student’s t-test (*P < 0.05; **P < 0.005).

**Figure 6:**
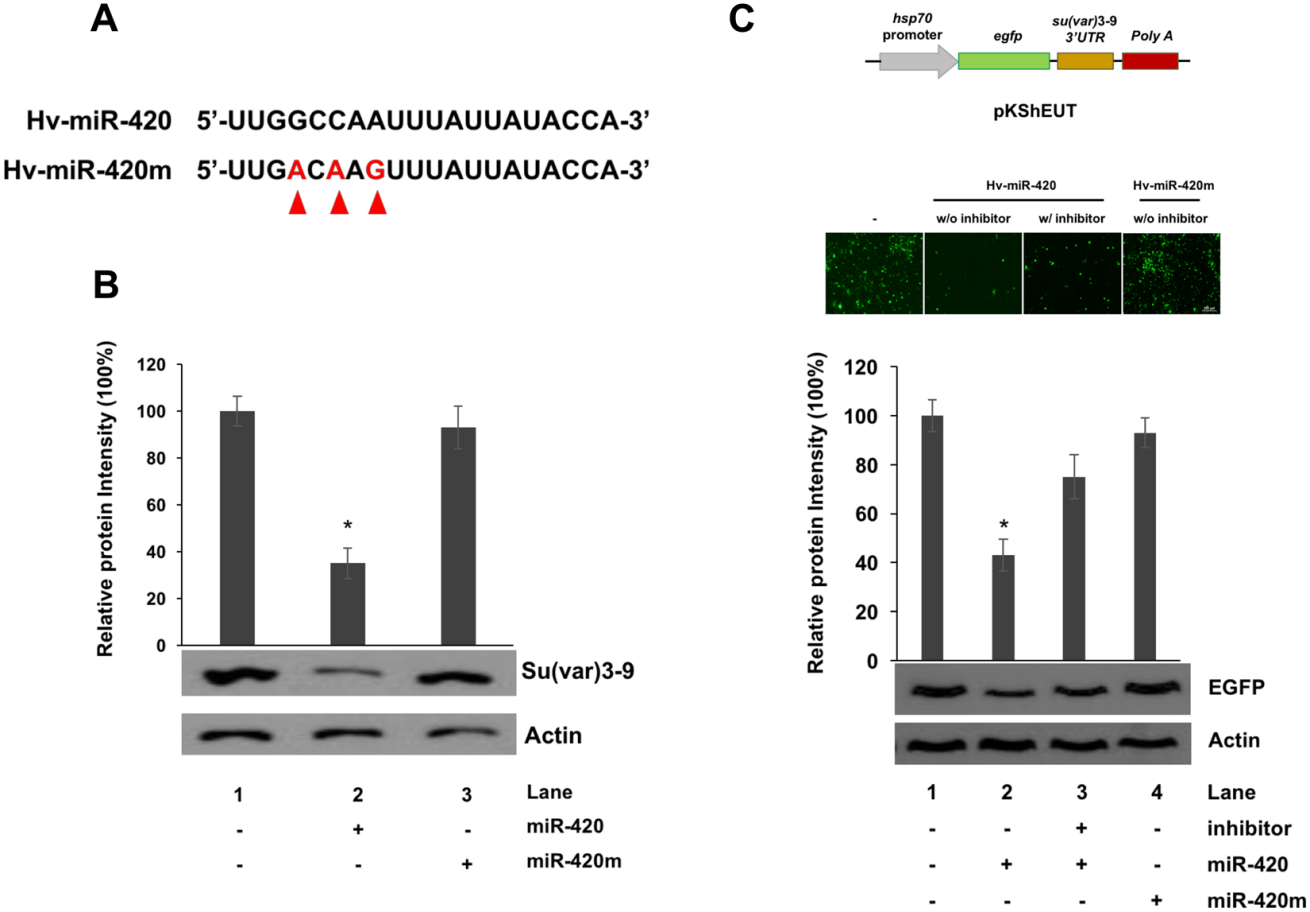
Base mutations in the seed region of hv-miR-420 is unable to suppress Su(var)3–9 expression. (**A**) Sequences of hv-miR-420 was shown, and miRNAs with mutations in the seed region were denoted as hv-miR-420m. The mutational substituted bases are indicated by arrows. (**B**) Expression levels of Su(var)3–9 in various treatments were analyzed by western blotting and the expression level of Actin were used as a loading control. (**C**) Reporter plasmids (pKShEUT) in which the 3′-UTR of *su(var)3–9* was fused to the 3′-end of the EGFP coding region, as indicated (top panels), were transfected into Sf-21 cells with miR-420 only, miR-420 plus miR-420 inhibitor or miR-420m. EGFP expression was detected at 0 or 48 hours after reporter plasmids (pKShEUT) transfection by fluorescent microscopy or western blotting. The samples were normalized to Actin, and the control group was set as 100%. At least three independent experiments were performed. P values were calculated using Student’s t-test (*P < 0.05).

Although the functions of several *pag1* miRNAs have been identified, the remaining miRNAs predicted from the *pag1* transcript need to be further studied^6^. To address this, the miRNAs predicted from *pag1* were mapped onto the *tip60* and *su(var)3–9* sequences (Fig. 3) and, based on the stem-loop PCR assay and the mode of action of miRNAs in general, Hv-miR-420 was selected for further experiments (Fig. 4). To evaluate the effect of miRNA-420 on *su(var)3–9*, Hv-miR-420 mimic was transfected into Sf-21 cells and it was found that 60% of *su(var)3–9* expression was suppressed 72 hours after transfection (Fig. 5A). To prove a direct relationship between Hv-miR-420 and suppression of *su(var)3–9*, Hv-miR-420 inhibitor was transfected into *pag1*-transfected cells (*pag1* transfected 24 hours prior to inhibitor transfection). Notably, the levels of *su(var)3–9* expression recovered to approximately 72% of the untreated control in contrast to 50% of the expression level in the *pag1*-transfected cells without inhibitor treatment (Fig. 5B). Hv-miR-420 expression levels in *Hz*NV-1-infected cells was measured and it was found to significantly increased after viral infection (Fig. 5C), where *su(var)3–9* protein levels was significantly decreased (Fig. 5D). Moreover, transfection of the Hv-miR-420 inhibitor increased Su(var)3–9 protein levels compared to *Hz*NV-1-infected cells without inhibitor (Fig. 5D). To confirm direct interaction between miR-420 and *su(var)3–9*, a mutant miR-420 which contained base mutations in its seed region was generated (designated miR-420m, Fig. 6A). Western blotting analysis (Fig. 6B) showed that the mutant miR420 did not affect the levels of Su(var)3–9 expression, suggesting that intact seed sequence was crucial for functioning. To assess whether the observed regulation was direct, the 3′ UTR of *su(var)3–9* was fused to the 3′-end of the *egfp* gene to generate an EGFP reporter plasmid. Co-transfection of this EGFP reporter plasmid with Hv-miR-420 significantly decreased EGFP levels, whereas co-transfection with the Hv-miR-420m did not affect EGFP expression (Fig. 6C). These results demonstrate that *su(var)3–9* expression was suppressed by increased Hv-miR-420 levels in both *pag1*-transfected and virus-infected cells.

### Hv-miR-420 is involved in regulating *Hz*NV-1 lytic infection

**Figure 7:**
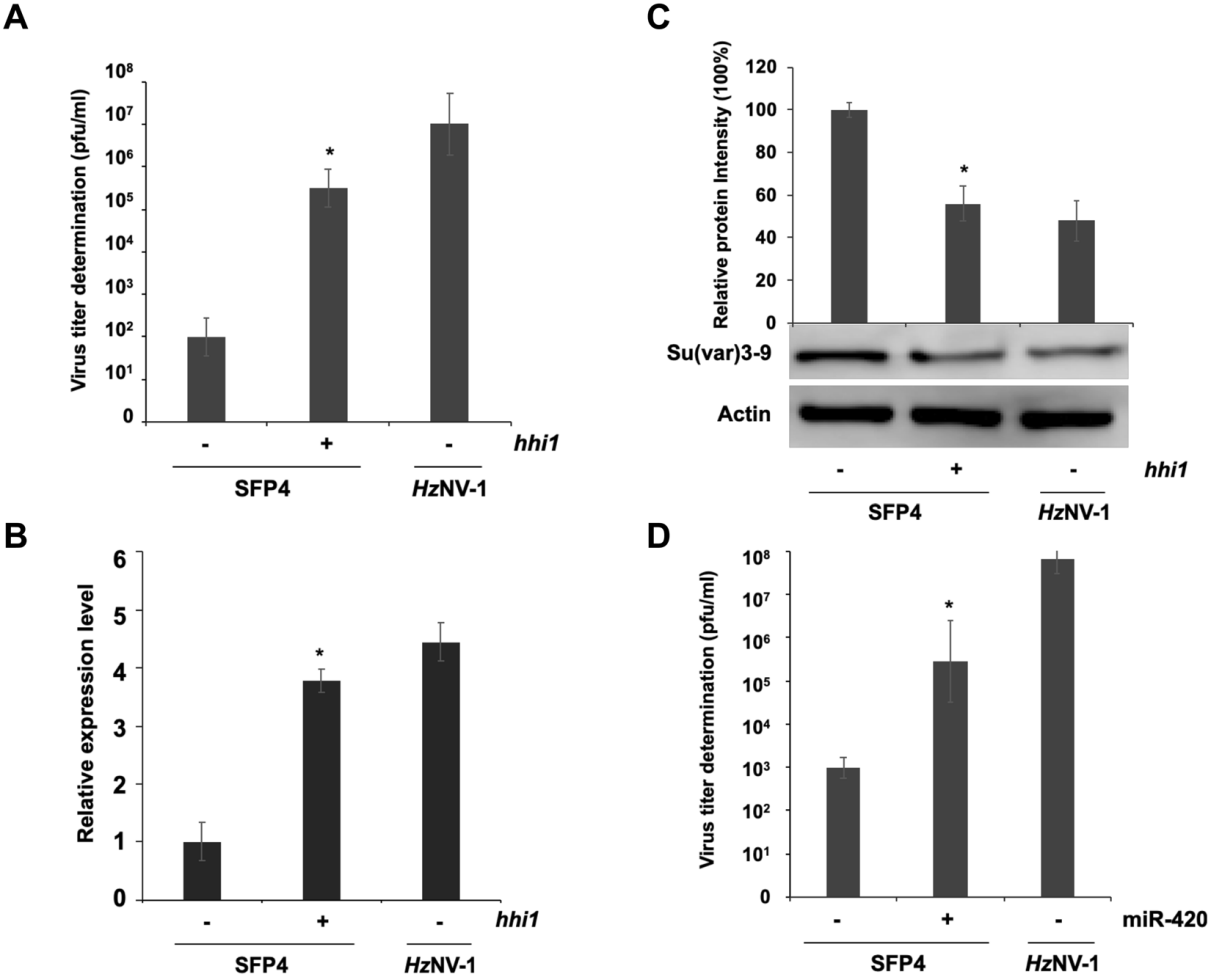
Induction of latent viral reactivation by miR-420. (**A**) Viral titers estimated in productively and latently infected cells. Latently infected cells were transfected with the plasmid pKShH1. (**B**) Stem-loop RT-qPCR confirmed miR-420 expression in *hhi1*-transfected SFP4 cells or *Hz*NV-1-infected Sf-21 cells. (**C**) Su(var)3–9 expression was downregulated in *hhi1*-transfected SFP4 cells. (**D**) Viral titers were estimated in latently infected cells. Latently infected cells were transfected with miR-420 mimic and control miRNA, and viral titers were determined at 72 hours post-transfection. Sf-21 cells were infected with the wild-type virus as a control. At least three repetitions were conducted for each group. P values were calculated using Student’s t-test (*P < 0.05).

Previous studies have found that the *pag1* transcript is the only *Hz*NV-1 viral transcript expressed during both lytic and latent infections^5^. In this study, changes in Hv-miR-420 expression at different infection stages were examined to evaluate whether Hv-miR-420 affected histone modification and altered infection stages. Virus titers in both latent infection cell lines (SFP4) and *Hz*NV-1-infected Sf-21 cells were analyzed following reactivation from latent stage induced by transfection of *hhi1* expression plasmid (Fig. 7A). Transfection of *hhi1* into HzNV-1-latently-infected SFP4 cells significantly increased Hv-miR-420 expression (Fig. 7B) but the expression of *su(var)3–9* was inhibited (Fig. 7C), indicating that Hv-miR-420 played essential roles in altering histone modification by latent viruses after reactivation. To assess whether Hv-miR-420 was able to stimulate latent viruses to enter lytic infection stage, Hv-miR-420 mimic was transfected into SFP4 cells and analyzed viral reactivation. The results showed significant cell lysis within 72 hours and increased virus titers after Hv-miR-420 transfection (Fig. 7D). These results demonstrated that Hv-miR-420 was able to induce latent viruses to enter lytic infection stage, possibly by contributing to activation of viral gene expression through inhibition of histone methylation.

## Discussion

The characteristics of *Hz*NV-1 are universal among mammalian viruses, especially HSVs, which share several common features with *Hz*NV-1. The noncoding latency-associated transcript (LAT) gene is highly expressed during HSV or HCMV latency and encodes a series of miRNAs^15,25,26,37,38^. Previous studies have focused on the interaction between viral miRNAs and viral genes, and it has been reported that LAT-encoded miRNAs not only inhibit the HSV IE genes ICP0 and ICP4 but also regulate histone modification^8,25,38,39^. However, these studies are still limited in insect viruses.

In this study, we discovered that the latent gene of *Hz*NV-1, *pag1*, was able to affect two insect histone transferases, *tip60* and *su(var)3–9*, at the beginning phase of infection (Fig. 2). *tip60* is involved in a variety of processes, such as DNA repair^40^, development^34^, and human virus association^41^. *su(var)3–9*, on the other hand, is a conserved histone transferase that also possesses multiple roles in animals and is related to the heterochromatin-associated HP1 protein together with H3K9 trimethylation^31,42^. We examined the expression of histone proteins containing modification in regions that are targets of the abovementioned histone transferases. Western blot analysis showed that trimethylation of H3K9 was significantly downregulated and that acetylation of H3 was upregulated after *pag1* transfection (Fig. 1). A possible explanation is that when methyltransferase expression decreases, resulting in reduced trimethylation of histones (H3K9), more histones are available for acetyl modification, leading to an increase in acetylated H3.

Acetylation of histones relaxes the structure of chromatin, typically resulting in gene activation. In contrast, methylation is reported to be associated with heterochromatin and gene silencing^43^. For this reason, most studies are inclined to relate methylation and deacetylation with latent infection. However, the ICP0 gene of HSV was found to be acetylated during quiescence^44^. Additionally, three early genes of KSHV are reported to have distinct histone modifications after KSHV primary infection. Indeed, relieving HDAC repression leads to activation of lytic genes in both HCMV and EBV^45,46^. This study indicates that repressive and active markers are simultaneously distributed across chromatin during latency^47^.

Similar to the LAT gene of HSV, *pag1* plays an important role during *Hz*NV-1 latent infection. Previous studies have shown that the *pag1* suppresses apoptosis induced by the lytic gene *hhi1*^48^ and that *pag1*-encoded miRNAs cause a switch from lytic infection to a latent infection^7^. Although the functions of several *pag1-*encoded miRNAs have been demonstrated, the functions of other predicted miRNAs still need to be confirmed^6^. To address this issue, miRNAs predicted from *pag1* were mapped onto the sequences of the *tip60* and *su(var)3–9* coding regions, and four candidates were chosen using universal reference miRNAs (Fig. 3). Based on stem-loop PCR and northern blot results, Hv-miR-420 was chosen for further experiments (Fig. 4). Two miRNAs (miR-795 and miR-475) (Fig. 4A,B) were expressed in untransfected cells, and there was no clear increase in their expression level upon *pag1* infection, suggesting that insect cells normally express low levels of these two miRNAs and they were not likely derived from *pag1* transcript. To determine the effects of Hv-miR-420 on *su(var)3–9*, we first transfected the miR-420 mimic into Sf-21 cells and found 60% suppression (Fig. 5A). To study opposing impacts, we then transfected miR-420 inhibitors, and nearly 30% of *su(var)3–9* expression was recovered after 72 hours (Fig. 5B). Taken together, these results show that the presence of miR-420 can regulate the expression levels of *su(var)3–9*. It has been reported that *pag1* is the only transcript expressed during both lytic and latent infections^6^. According to a previous report, the *pag1* transcript yields several miRNAs, some of which were shown to maintain latency^7^. The ability of miR-420 to stimulate a virus toward lytic infection may be due to differential expression of *pag1*-derived miRNAs during lytic and latent stages. Our preliminary results show relatively low miR-420 expression in latently infected cells, suggesting that the generation of miR-420 was downregulated. Investigation of how the expression of *pag1*-derived microRNAs are regulated is currently underway.

The difficulty in establishing a stable HSV-latently-infected cell line has hampered the progress of HSV latent infection research. Nonetheless, a permanent cell line of *Hz*NV-1 latent infection, namely, SFP4 cells, is available^49^ and the results of *pag1* transfection could be validated in the context of HzNV-1 latent infection. Indeed, we performed experiments with *pag1* to eliminate other variables correlated with *Hz*NV-1. Our discovery of an epigenetic pathway that had been modified by Hv-miR-420 indicates that some variables regarding Hv-miR-420 and HMT *su(var)3–9* in the dual stages of *Hz*NV-1 may need to be taken into consideration in the future. According to an earlier study, *pag1* is detectable in both lytic and latent stages^7^. Under these circumstances, we may be able to utilize a reactivating virus from SFP4 cells to compare different expression levels of Hv-miR-420, *su(var)3–9*, and trimethylation H3K9, which can help in determining whether this microRNA has a crucial role in reactivating *Hz*NV-1.

**Figure 8:**
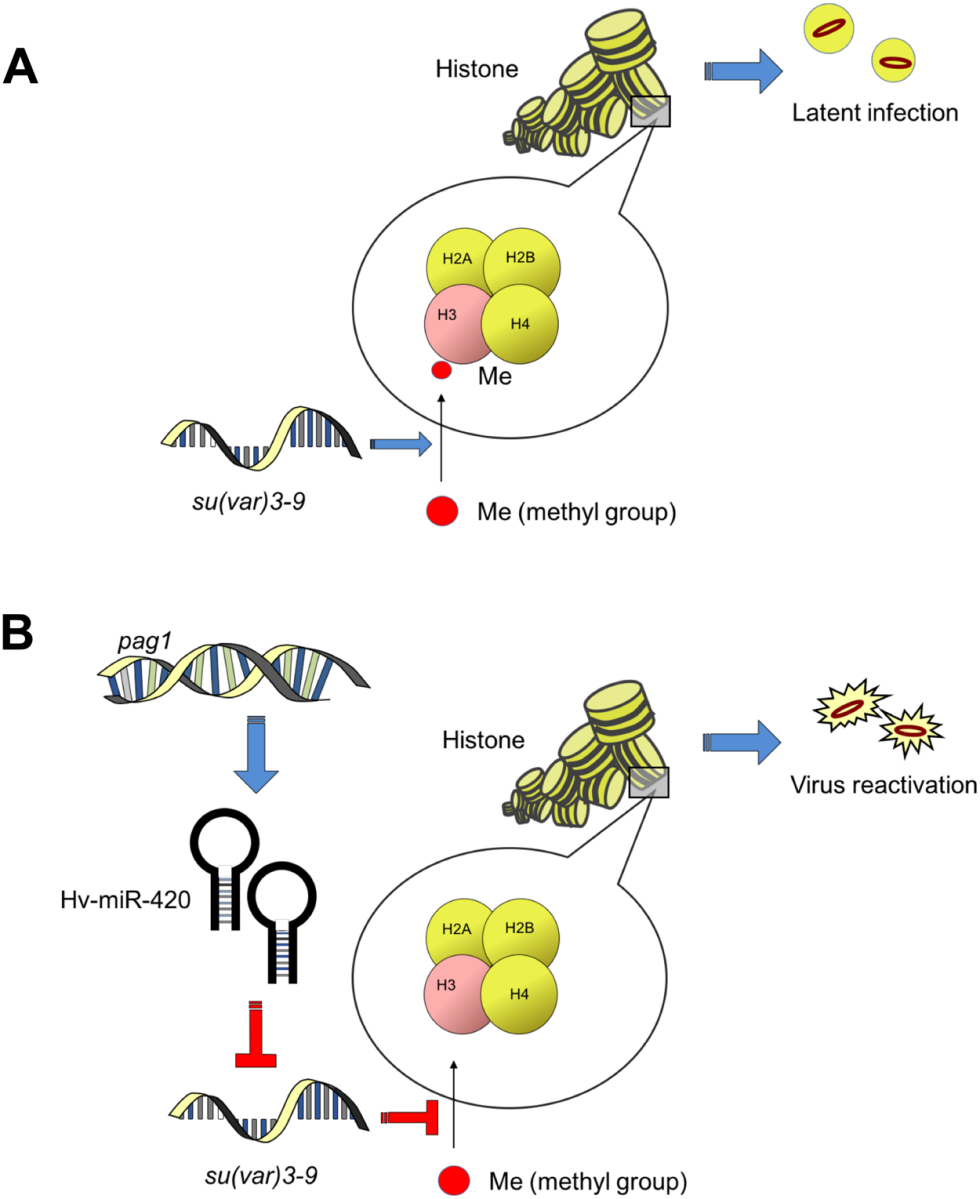
A model of the establishment of lytic infection via miR-420-mediated suppression of histone methylation. (**A**) Without miR-420. (**B**) With miR-420.

Previous studies on human latent viruses, e.g., HSV^50^, KSHV^21^, HBV^51^ and HCMV^52^, found that host HATs are affected by these viruses. In contrast, few studies have investigated interaction between viral miRNAs and histone modification. Our research presented herein addressed this interaction and revealed that Hv-miR420, a miRNA produced from the *pag1* gene, regulates host HMT *su(var)3–9* to suppress H3K9 trimethylation. Surprisingly, a tentative explanation is that the decrease in H3K9 trimethylation during latency is a sign of reactivation (Fig. 8). The physiological significance and *in vitro* tests need to be pursued to further support and verify our findings. This work on the functions of latent miRNAs from an epigenetic perspective provides a better understanding of insect latent viruses.

## Materials and Methods

### Cell culture and virus

*S*. *frugiperda* IPLB-Sf-21 cells were cultured in TC-100 insect cell medium with 10% fetal bovine serum (FBS) at 26 °C (Gibco BRL)^53^. The SFP4 cell line was derived from latently infected Sf-21 cells^4,5^. *Hz*NV-1 titers were calculated using TCID_50_^54^ and quantitative PCR (qPCR)^55^.

### Plasmid *DNA* construction and transfection

The coding regions of *hhi1* and *pag1* were previously inserted into the vector pKSh to construct pKShH1 and pKShP1^7^. To clone the 3′-UTR of *su(var)3–9* (primer: 3–9 3UTR-F: ACGCACGCTCATTCTGACACACGC; 3–9 3UTR-R: TTTACAATCTTATTACATTTAC) into an EGFP reporter vector (pKShE)^5^, the *su(var)3–9*-UTR fragment was inserted into the *EcoR*I site after the stop codon of the EGFP gene (pKShEUT). A total of 2 × 10^5^ Sf-21 cells were seeded into a 24-well culture plate (Corning, Action, MA) and transfected with 0.5 μg of plasmid DNA using Cellfectin (Invitrogen, Carlsbad, CA) following the manufacturer’s protocol (Gibco BRL). EGFP expression was observed using fluorescent microscope at 2 days-post transfection. Samples were collected at 48 hours post-transfection in triplicate.

### Western blotting

Proteins were isolated with 40 μl of RIPA buffer (Thermo Fisher) per well in a 24-well culture plate, and the samples were immediately stored at −80 °C until use. Western blotting was carried out using rabbit polyclonal antibodies detecting acetyl-H3 (Millipore, 1:4500), trimethyl-H3K9 (Millipore, 1:500), su(var)3–9 (Abcam, 1:4000) and EGFP (Millipore, 1:5000). Normalization was performed via detection of actin levels using a mouse polyclonal antibody (Millipore, 1:2500). All bands were analyzed and quantified using AlphaView SA.

### RT-PCR and qPCR

RNA samples were collected using 100 μl of GENEzol^TM^ reagent per cm^2^ of culture dish with a GENEzol^TM^ TriRNA Pure Kit (Geneaid) and stored at −20 °C. The eluted RNA was reverse transcribed using a PrimeScript^TM^ RT-PCR Kit (TaKaRa), and cDNA was synthesized from 800 ng of RNA for each sample. Quantification of gene expression was carried out by adding 2 μl of cDNA to 2 × SeniFAST SYBR^®^ Hi-ROX Mix (BIOLINE) using a 56 °C annealing temperature. PCR was carried out using the following primers:

*Actin* F: TCAACCCCAAGGCCAACAGAGA

*Actin* R: GAGGCTCCTGGGAATTTTTC

*Tip60* F: CGCGAAATGGTAACAAACAG

*Tip60* R: TGGAGAGCCACATAACAACTG

*Su(var)3–9*F: CGCCTGTCGGACTCAGTTAT

*Su(var)3–9*R: GAGGCTCCTGGGAATTTTTC

### Computational prediction of viral miRNAs

*pag1* miRNA prediction was implemented according to the full-length gene sequence of *pag1* (NC_004156.1), which was obtained from the National Center for Biotechnology Information. The prediction was conducted using the miRNA prediction database (Vir-Mir) (http://alk.ibms.sinica.edu.tw). The miRNAs with high match scores chosen for analysis are listed in Table 1.

### Stem-loop real-time PCR and northern blotting

Sf-21 cells transfected with pKShP1 at 0 and 48 hpt were used for extraction of total RNA using TRIzol® reagent (Invitrogen) following the manufacturer’s instructions. The method for detecting mature miRNAs with specific primers was described in a previous study^56,57^. The miRNA primers used for RT-PCR and real-time qPCR were as follows:

Hv-miR-420 RT: 5′-GTCGTATCCAGTGCAGGGTCCGAGGTATTCGCACTG

GATACGACTGGTAT-3′

Hv-miR-420 F: 5′- CACGCATTGGCCAATTTA-3′

Hv-miR-150 RT: 5′-GTCGTATCCAGTGCAGGGTCCGAGGTATTCGCACTG

GATACGACCTTGTA-3′

Hv-miR-150 F: 5′- CACGCAATCCATGATTAA-3′

Hv-miR-795 RT: 5′-GTCGTATCCAGTGCAGGGTCCGAGGTATTCGCACTG

GATACGACCACGGA-3′

Hv-miR-795 F: 5′- CACGCAGTCTTTTTGCAG-3′

Hv-miR-475 RT: 5′-GTCGTATCCAGTGCAGGGTCCGAGGTATTCGCAC

TGGATACGACACTTTA-3′

Hv-miR-475 F: 5′- CACGCACACCACCATTCG-3′

Reverse primer: 5′-CCAGTGCAGGGTCCGAGGTA-3′

Total small RNA samples from *pag1*-transfected and *Hz*NV-1-infected cells were isolated using a mirVanaTM miRNA isolation kit (Ambion) according to the manufacturer’s instructions. For northern blot analysis of small RNAs, 1 µg of small RNA sample and radio-labeled Decade Marker (Ambion) were fractionated by 15% denaturing polyacrylamide gel electrophoresis (PAGE) (acrylamide∶bis ratio, 19∶1) containing 8 M urea in 0.5 × TBE buffer. RNAs were transferred by electroblotting to a Hybond-N + nylon transfer membrane (GE Healthcare) and UV cross-linked. RNA oligonucleotides (Integrated DNA Technologies) carrying the reverse complementary sequence for candidate miRNAs or let-7a were end-labeled with DIG (MP Biomedicals, Irvine, CA) to high specific activity. Hybridizations and washes were carried out using DIG hybridization buffer according to the manufacturer’s directions (Roche).

### Mimic and inhibitor miRNA transfection

The Hv-miR-420 mimic was synthesized by the MDBio Incorporation, and inhibitor was produced by GenePharma Incorporation. The mimic (50 nM) or inhibitor was transfected into Sf*-*21 cells using 1.5 μl of Lipofectamine^TM^ RNAiMAX following the manufacturer’s protocol (Invitrogen). The sequences of the Hv-miR-420 mimic, Hv-miR-420m and inhibitor are 5′-UUGGCCAAUUUAUUAUACCA-3′; 5′-UUGACAAGUUUAUUAUACCA-3′ and 5′-UGGUAUAAUAAAUUGGCCAA-3′, respectively.

### Statistical analysis

The qPCR *C*t values for samples treated with plasmids were normalized to those of actin with the comparative *C*t method^58^. Each group of experimental data was selected for comparison with the control groups using a single tail and type 1 t-test. The statistics used in this data analysis were performed with the T-TEST function in Microsoft Excel. P values are indicated in figures, with (*) representing the level of significance.
